# Health and environmental impact assessment of landfill mining activities: A case study in Norfolk, UK

**DOI:** 10.1016/j.heliyon.2022.e11594

**Published:** 2022-11-17

**Authors:** Mohammed Zari, Richard Smith, Charles Wright, Rebecca Ferrari

**Affiliations:** a University of Nottingham, Faculty of Engineering, Chemical and Environmental Engineering Department, Coates Building, University Park, Nottingham NG7 2RD, United Kingdom; b Department of Environmental Science, Faculty of Meteorology, Environment and Arid Land Agriculture, King Abdulaziz University, Jeddah, Saudi Arabia; c Industrial Chemicals Ltd, Titan Works, Hogg Lane, Grays, Essex RM17 5DU, United Kingdom; d Norfolk County Council, County Hall, Martineau Ln, Norwich NR1 2DH, United Kingdom

**Keywords:** Heavy metals, Municipal solid waste, Landfill mining, Pollution assessment, Human health risk assessment

## Abstract

The release of fine particles during mechanical landfill mining (LFM) operations is a potential environmental pollution and human health risk. Previous studies demonstrate that a significant proportion (40–80% wt) of the content of fine soil-like materials within the size range <10 mm to <4 mm recovered from such operations originate from municipal solid waste (MSW) landfills. This study evaluates the potential health risks caused by emissions from LFM activities. MSW samples recovered from the drilling of four different wells of a closed UK landfill were analysed for physical, chemical, and biological properties to determine the extent of potential contaminant emissions during LFM activities. The results show that fine particles (approximately ≤1.5 mm) accounted for more than 50% of the total mass of excavated waste and contained predominantly soil-like materials. The concentrations of Zn, Cu, Pb, Cd, As, and Cr exceed the permissible limits set by the current UK Soil Guideline Values. The highest geoaccumulation index and contamination factor values for Cu were 2.51 and 12.51, respectively, indicating a moderate to very high degree of contamination. Unsurprisingly, the pollution load index was >1, indicating the extent of pollution within the study area. The hazard quotient values indicated high exposure-related risks for Pb (16.95), Zn (3.56), Cd (1.47), and As (1.46) for allotment land use and As (1.96) for residential land use. The cancer-related risk values were higher than the acceptable range of 1.0 × 10^−6^ to 1.0 × 10^−4^. The cancer risk factor indicated that Cr and As were the major human health risk hazards.

## Highlights

• Potentially toxic elements and organics associated with waste fine fractions.
• Novel method for assessing potential human health risk of heavy metals achieved.
• Landfill poses major risk to human health and environment if LFM occurs.
• Pb highest contributor to the non-carcinogenic risk.
• Cr most prominent metal with respect to carcinogenic effect.

## Graphical abstract

**Figure:**
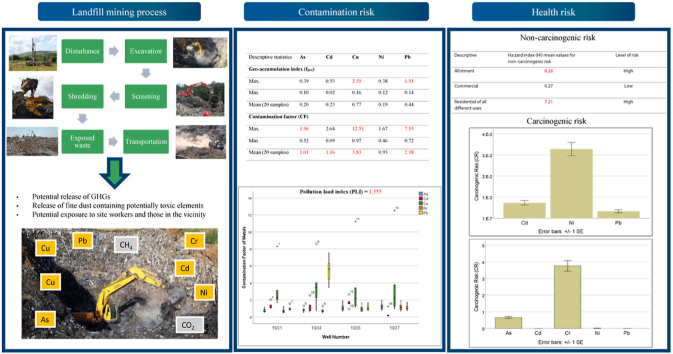

## 1 Introduction

Dust emissions, particularly potentially toxic elements released into the atmosphere, associated with mining are a pollution-related cause of human health problems (Adewumi and Laniyan, 2020; Guo et al., 2021; Li et al., 2017; Soltani et al., 2021; Tepanosyan et al., 2018). Likewise, landfill mining (LFM) activities, including excavation, shredding, screening, and equipment handling, also lead to the release of potentially harmful particulate emissions into the environment (Ilse et al., 2018; Pastre et al., 2018) as short-term episodic emissions during operational periods. Despite the increasing interest in LFM and its development during the last two decades, the release of dust from mining and landfill mining activities into environmental media remains a human health issue of concern (Adelopo et al., 2018; Entwistle et al., 2019; Tang et al., 2015; Zhou et al., 2020), especially where historic landfills have the potential to cause contamination (González-Martínez et al., 2019). LFM involves the excavation of waste from a landfill site following a prolonged period of closure, usually measured in decades, during which time the site has stopped receiving waste (Hogland et al., 2004; Hull et al., 2005; Krook et al., 2012; Rodriguez et al., 2018; Somani et al., 2018) and active waste degradation processes are very much diminished. Owing to the growing interest in landfill mining and reclamation activities for materials and energy recovery (Pastre et al., 2018), or simply for site redevelopment, an evaluation of the extent of heavy metal enrichment and related health risks is required to avoid further heavy metal deposition in the environment (Adelopo et al., 2018) and prevent unacceptable health risks. Waste fractions generally consist of decomposed organic materials, mineral waste, and heavy metals (López et al., 2018), which can become airborne through various processes during the LFM (Ilse et al., 2018). Heavy metals are of great concern because, unlike organic pollutants, they remain unaffected during the degradation of waste, thereby having adverse impacts on living organisms (Esakku et al., 2003; Hogland et al., 2014; Jain et al., 2005; Mehta et al., 2019; Ngole-Jeme and Fantke, 2017). Some metals such as, Cd, Cr, and Pb are well known to be toxic to human health, even at very low concentrations (Adewumi and Laniyan, 2020; Csavina et al., 2012; Padoan et al., 2020), and can pose carcinogenic risks (Bhatti et al., 2020; Gujre et al., 2021; Kamunda et al., 2016; Thongyuan et al., 2020). Exposure to such heavy metal concentrations may lead to numerous health problems, particularly in susceptible individuals, including the elderly and children (Briki et al., 2017; Csavina et al., 2012; Dubey et al., 2012; Kamunda et al., 2016; Stewart, 2019). Fine fractions of soil-like materials within size range of <10 mm to <4 mm can account for up to 40–80 wt.% of the total waste excavated (Chandana et al., 2020; Datta et al., 2020; Jani et al., 2016; Kaartinen et al., 2013; Masi et al., 2014). This was evidenced by a recent investigation of nine landfill sites located across the UK that showed that fine soil-like material accounted for 30–74% (w/w) of the total waste excavated (Wagland et al., 2019). Finer fractions are more problematic because they are more mobile in the environment and particulates <2.5 μm can penetrate bronchioles, causing serious lung damage (Guan et al., 2016; Shou et al., 2019; Wang et al., 2022; Xing et al., 2016; Xu et al., 2018). Large quantities of these soil-like fractions in landfills is attributable to the application of daily soil cover, deposition of construction and demolition waste, and humification of organic matter (Somani et al., 2018, 2019). Humic materials are formed from the biodegradation of organics within waste (Wagland et al., 2019).

A critical review of studies on LFM revealed that research has primarily focussed on material and energy recovery (Dino et al., 2018; Gutiérrez-Gutiérrez et al., 2015; Mehta et al., 2020; Pecorini and Iannelli, 2020). However, there is a fundamental lack of understanding of how these activities could affect the environment and human health (Frändegård et al., 2013; Nguyen et al., 2018; Padoan et al., 2020). Therefore, this study considered such environmental and human health impacts. Existing landfill site sampling programs were used to inform the characterisation of physical, chemical, and biological properties of landfilled waste. Characterisation results were then utilised to obtain pollution and health impact indices and other key indicators.

## 2 Material and methods

### 2.1 Site selection and description

Landfilling in the UK before the introduction of modern engineering and regulatory standards, the current environmental permitting regime, and implementation of the Landfill Directive, was largely carried out by local authorities and private sector companies. The site selection for this study was conducted by identifying a site that is a typical representative of hundreds of landfills of this type that remain as historic deposits in the UK, largely filled during the 1970s, 1980s, and the early 1990s.

**Figure 1:**
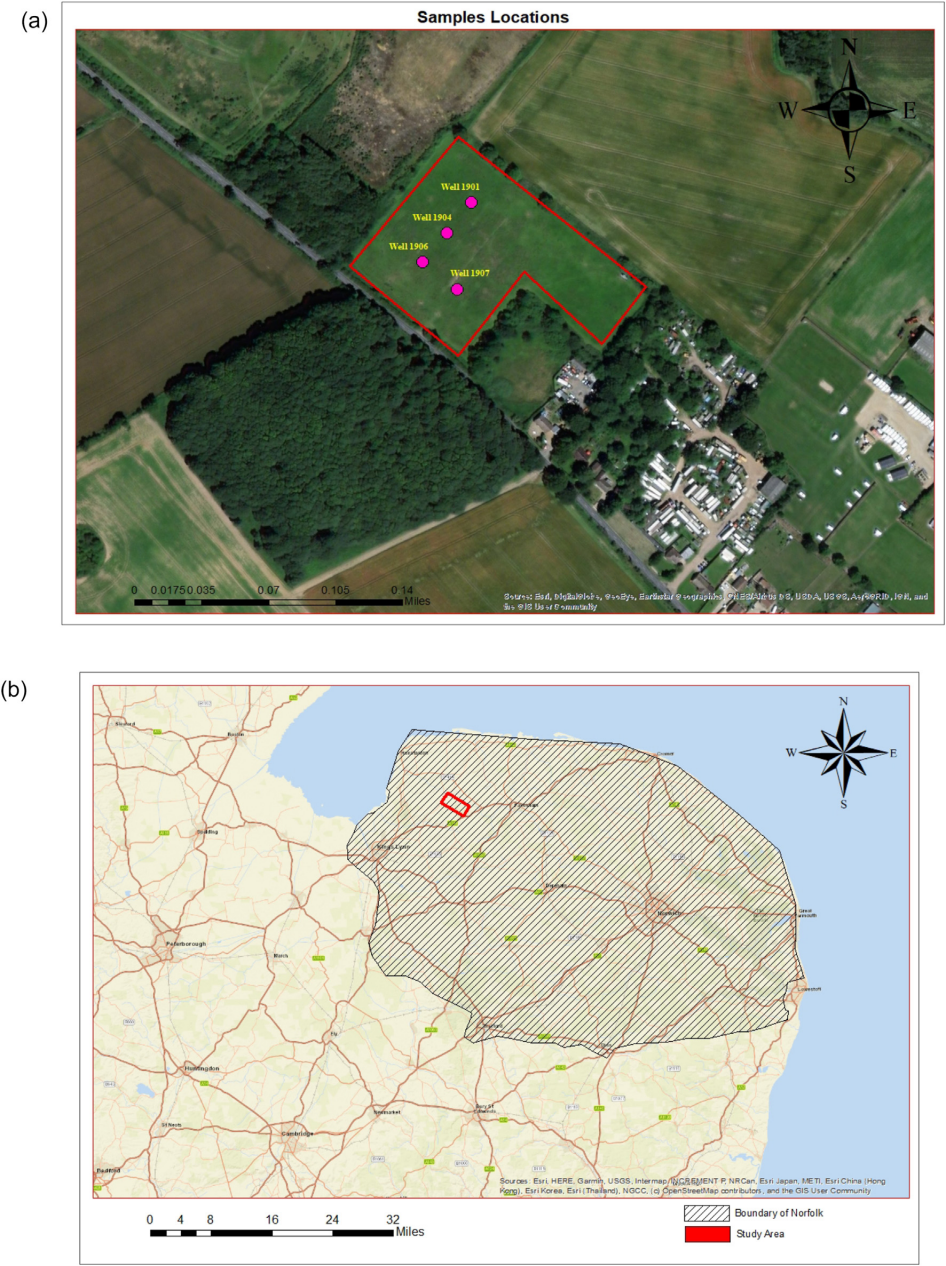
(a) Aerial photograph of the study area marked with a red boundary and sample locations illustrated by pink circles with corresponding well numbers. (b) Map of the site location illustrating the boundary of Norfolk and the study area marked with a red boundary.

Docking Common Landfill is located in Norfolk, a county in East Anglia, England (Figure 1 a and b). The site is a historical mineral working (sand and gravel pit), which was subsequently utilised as a local authority landfill by Norfolk County Council. The site was operational from 1978 to 1986, is approximately 10 m deep, and has a surface area of 3.12 hectares (approximately 7.71 acres). The landfill was unlined (non-engineered) and an engineered geo-synthetic clay liner cap was installed in 1998 to prevent rainfall infiltration and leachate generation as well as enabling intermittent landfill gas extraction. The site predominantly accepted municipal solid waste (MSW) with commercial and industrial waste, and it was operated and regulated on a co-disposal basis under the Control of Pollution Act (1974). Landfills consist of a complex mixture of organic and inorganic waste. Figure 1 a and b obtained using ArcMap 10.4.1, which shows the study area/sampling locations and boundary of Norfolk/the study area, respectively.

### 2.2 Excavation and sample processing

Nearly 40 kg of MSW was collected from four different wells, numbered 1901, 1904, 1906, and 1907 (10 kg each), using a rotary drilling rig with a depth ranging from 7–8 m. The samples were placed in thick-labelled plastic bags and sealed firmly to prevent any loss. Samples were transported in a rigid plastic box for transfer to the laboratory and were subsequently stored in refrigerated conditions at 4 °C until preparation for Total Organic Carbon (TOC) analysis. Representative samples were obtained through coning and quartering. Bulky non-biodegradable materials, such as plastic, metal, paper, and textile, were removed from the recovered MSW samples by manual sorting for homogenisation of the samples for analytical purposes. Approximately 20 kg of representative samples were obtained from coning and quartering (5 kg per well). Using a riffle sample splitter, these 5 kg samples from each well were further subsampled to approximately 1 kg each. The samples were oven-dried at 105 °C for 3 days to enable inorganic analysis. Finally, samples were subjected to mechanical sieving to divide them into selected size fractions.

### 2.3 Analysis

#### 2.3.1 Heavy metals (elemental analysis)

A total of 20 samples from the four wells (five per well) of different size fractions (≤0.106 mm) were analysed for heavy metals. The analyses were performed using an Agilent 5110 VDV Inductively Coupled Plasma Optical Emission spectroscopy (ICP-OES) equipped with a sea-spray nebuliser and concentric spray chamber and controlled using the ICP Expert software. ICP-OES is more robust for analysing solid waste because samples may contain suspended solids (Thermo Fisher Scientific, 2015). Approximately 0.5 g was used for each sample. Digestion was performed using 9 ml of concentrated nitric acid and 3 ml of hydrochloric acid, according to the elemental combination considered to achieve the highest possible quantity of extracted metals. The samples were then placed in a microwave leach using concentrated acids, as described in the United States Environmental Protection Agency (USEPA) method 3051A. The leachates were then diluted to 100 ml using 18.2 MOhm water and filtered through a 0.45-μm filter prior to analysis. Digestion analysis was carried out in two batches through the microwave for ICP-OES analysis. Each batch contained a reagent blank to test for contamination and carryover. Each batch contained a homogeneous soil sample to ensure repeatability. Multicomponent calibration standards were set according to Romil's single-element certified standards; thus, dilution was performed in the sample matrix (9% v/v nitric acid, 3% v/v HCl). K, Ca, and Na were also added to mimic the ionic strength of the samples. The presence of Fe in the samples interfered with the quantification of Cd and Pb; therefore, a correction called the fast automated curve-fitting technique (FACT) was applied to deconvolute the spectra prior to quantification. Two wavelengths were reported for each element for confirmation purposes. In this study, the following metals were analysed: Pb, Cd, Zn, Cu, Cr, Co, As, Ni, Ba, and Mn, which were selected as being of greatest concern in European and American communities (Abu-Daabes et al., 2013; Cortés et al., 2021).

#### 2.3.2 Total organic carbon (TOC)

TOC is the total carbon present as organic molecules (Jovanov et al., 2018). TOC is a more direct expression of the total organic content compared to other similar parameters (Abu-Daabes et al., 2013). Methane is a potent greenhouse gas produced by organic waste degradation (García et al., 2016) and can pose a risk to local residents because of its flammability and explosivity (Mou et al., 2014; Rong et al., 2017; Weng et al., 2015). Knowing the organic content helps determine the state of degradation of landfilled waste, which is a critical parameter for determining the suitability of a site for landfill mining (Pecorini and Iannelli, 2020). Ten samples of the fine fractions less than 0.106 mm from wells 1901 and 1904 (five from each well) were analysed using a LECO CHN628 TOC analyser.

### 2.4 Statistical analysis

Statistical analysis was performed using the Statistical Package for Social Science (SPSS) software (version 27). A one-way analysis of variance (ANOVA) followed by least significant difference (LSD) post hoc analysis was used to estimate statistically significant differences among the four wells and to compare the heavy metal contents of the MSW samples. Pearson's correlation (r) analysis using a 2-tailed test was used to identify correlations between various variables in the MSW samples. Box and whisker plots were produced to display the range of environmentally available heavy metal concentrations in the four different wells.

### 2.5 Heavy metal pollution assessment

Generally, pollution indicators are the most efficient and suitable tools for the assessment of soil heavy metal pollution (Doležalová Weissmannová et al., 2019); the selection of these is discussed below.

#### 2.5.1 Geoaccumulation index

The geoaccumulation index (I_geo_) was used to examine the contamination level of landfill precursors affected by metals. It is a geochemical criterion (unitless) coined by (Muller, 1969) and has been widely employed in European research on trace metals (Li et al., 2014). It is calculated using the following equation (Gujre et al., 2021) (1):

(1) $$I_{geo}={\mathrm{Log}}_{2}\left(\frac{C_{n}}{1.5B_{n}}\right)\text{,}$$

where Cn is the measured concentration of heavy metal analysed in the landfill precursors and Bn is the normal background concentration in English soils, as reported by the British Geological Survey (BGS) (Johnson et al., 2012). A constant of 1.5 was introduced to minimise potential variations in background values, referred to as lithogenic variations (Aiman et al., 2016; Hassaan et al., 2016). Classification of the I_geo_ pollution levels is presented in (Table S1) (Muller, 1969; Rahman et al., 2012; Tang et al., 2015).

#### 2.5.2 Contamination factor

The contamination factor (CF) indicator represents the anthropogenic contribution of heavy metal pollution and is commonly used as a measure for landfill precursor pollution assessment (Adelopo et al., 2018; Pandey et al., 2016). The CF was obtained by dividing the concentration of heavy metals in the waste samples by their background concentrations (Chen et al., 2015). Reference concentrations considered were obtained from the BGS (Johnson et al., 2012) and the CF (unitless) was calculated according to Eq. (2) (Hakanson, 1980):

(2) $$CF=\frac{C_{i}}{B_{i}}\text{,}$$

where Ci is the concentration of the analysed heavy metal and Bi is the geochemical background value of that metal. The pollution levels of the CF were divided into seven classes, numbered 0 through to 6 (Table S1) (Doležalová Weissmannová et al., 2019; Pandey et al., 2016; Rahman et al., 2012).

#### 2.5.3 Pollution load index

To assess the overall pollution, the pollution load index (PLI) provides an established approach for calculating the accumulation of heavy metals in samples (Kowalska et al., 2018; Wang et al., 2020). The PLI (unitless) can be obtained by calculating the geometric mean of the CFs of each element analysed (Tomlinson et al., 1980), as follows (Sharifi et al., 2016; Thongyuan et al., 2020):

(3) $${PLI=\left({CF}_{1}\;X\;{CF}_{2}\;X\;{CF}_{3}\;X\ldots X\;{CF}_{n}\right)}^{1/n}\text{,}$$

where *n* is the number of analysed heavy metals and CF is the contamination factor of each metal. A PLI value > 1 indicates the presence of pollution, whereas no pollution load is indicated by a value < 1 (Pandey et al., 2016; Thongyuan et al., 2020; Tomlinson et al., 1980).

### 2.6 Potential human health risk of heavy metals

A human health risk assessment is used to assess the potential impacts of chemical exposure in contaminated environmental media on human health (Li et al., 2014; Reyes et al., 2021). It is extensively used for estimating the health effects of heavy metals as a result of exposure to these chemicals (Doležalová Weissmannová et al., 2019). Quantification of heavy metals has been categorised by the (USEPA, 2002) as being non-carcinogenic or carcinogenic in human health risk assessments (Kamunda et al., 2016; USEPA, 2002). The exposure of humans to heavy metals from soil is estimated through three main exposure routes: ingestion of substrate dust particles, inhalation of suspended dust particles through mouth/nose, and dermal contact/absorption of heavy metals in particles adhered to exposed skin, according to the recommendations and methodology of the (USEPA, 2002) (Adelopo et al., 2018; Gujre et al., 2021; USEPA, 2002). The non-carcinogenic risk effect is typically characterised by the hazard quotient (HQ), which is defined as the ratio of the average daily intake to the toxicity threshold value (also referred to as the reference dose) of a chemical for the same exposure (Thongyuan et al., 2020; Yan et al., 2022). It is also characterised by the hazard index (HI), which estimates the overall potential for non-carcinogenic effects (Doležalová Weissmannová et al., 2019). Both the HQ and HI are unitless, expressed as an individual's likelihood of experiencing adverse effects. The equation used in this study was based on the recommendations provided by the Environment Agency, 2009b, Environment Agency, 2009a (Hosford, 2009). In practice, when soil guideline values (SGVs) exist for a metal, the HQ and HI can be estimated by dividing the soil concentration of each contaminant by its SGVs and summing the results (Hosford, 2009). The derivation of SGVs was calculated based on all the exposure routes. The HQ of each chemical was determined using Eq. (4):

(4) $$HQ\;\left(non-carcinogenic\right)=\frac{C_{c}}{SGV}\text{,}$$

where Cc is the contaminant concentration of each element and SGV is the soil guideline for the corresponding element. The HQ represents the non-carcinogenic risk from individual heavy metals, whereas the HI is the sum of the hazard quotient and indicates the cumulative non-carcinogenic risk. The HI was determined according to Eq. (5):

(5) HI(non−carcinogenic)=∑HQ,

HQ and HI values < 1 indicate a lack of adverse non-carcinogenic effects on health, whereas if HQ and HI > 1, non-carcinogenic adverse health effects may occur (Gujre et al., 2021), and the likelihood of effects increases as the HQ/HI value increases. The carcinogenic risk (CR) and lifetime cancer risk (LCR) were calculated using Eqs. (6) and (7), respectively, which express the likelihood of developing cancer in a lifetime due to potential carcinogen exposure (unitless).

(6) CR(carcinogenicrisk)=Cc×SF,

(7) LCR=∑CR,

where Cc is the contaminant concentration of each element analysed and SF is the cancer slope factor identified by (USEPA, 2002). The values for As, Cd, Pb, Cr, and Ni are 20.26, 6.3, 0.0085, 42, and 0.84 mg/(kg‧day), respectively (Adimalla and Wang, 2018; Chen et al., 2015; Ferreira-Baptista and De Miguel, 2005; USEPA, 2002). The cancer SF directly converts the estimated daily intake of an average toxin over a lifetime of exposure to the incremental risk of developing cancer (Li et al., 2014). The LCR is the summation of CR values and indicates the overall risk. Values above 1 × 10^−4^ are considered unacceptable and indicate significant health effects, whereas values below 1 × 10^−6^ indicate nonsignificant health effects (Gujre et al., 2021). Values ranging from 1 × 10^−4^ to 1 × 10^−6^ are generally considered tolerable (Fryer et al., 2006; Hu et al., 2012; Li et al., 2014; Tenebe et al., 2018).

## 3 Results and discussion

### 3.1 Municipal solid-waste characterization

#### 3.1.1 Concentration of heavy metals in MSW

The main heavy metal content is reflected in the solid form of the waste resulting from the interaction between heterogeneous landfilled waste, local landfill management (e.g., top layer), climatic conditions, and degradation activities (Adelopo et al., 2018; Holm et al., 2002). In this regard, some studies have demonstrated that the concentrations of heavy metals in solid waste samples are significantly higher than those in landfill leachates (Øygard et al., 2004; Xiaoli et al., 2007). During biodegradation, the metal content increased with volume reduction (Esakku et al., 2003). This means that older landfills have higher concentrations of heavy metals than younger landfills because of the transformation processes of fresh MSW over time (Jain et al., 2005; Quaghebeur et al., 2013) that accumulates substances. Table 1 presents the heavy metal concentrations of the four wells for different size fractions. In the fractions, the concentrations of the metals followed the order Zn > Mn > Pb > Cu > Ba > Cr > Ni > As > Co > Cd. Previous investigations on the characterisation of particle size fractions associated with heavy metals showed that the accumulation of heavy metals is maximal for fine fractions because of the high specific surface area of fines fractions (Burlakovs et al., 2018; Wei et al., 2015; Wolfsberger et al., 2015; Yao et al., 2015). Therefore, the heavy metals were analysed for fine fraction samples of <0.106 mm from the four wells. In the Appendix, Table A1 shows the descriptive statistics of the metals within the four wells.

**Table 1:** Selected heavy metal concentrations of the four wells for various size fractions.

Well number	Particle size (μm)	As (mg/kg)	Ba (mg/kg)	Cd (mg/kg)	Co (mg/kg)	Cr (mg/kg)	Cu (mg/kg)	Mn (mg/kg)	Ni (mg/kg)	Pb (mg/kg)	Zn (mg/kg)
**1901**	<38	37.1	273.9	2.0	16.5	172.7	514.1	2039.2	51.7	285.1	2205.6
**1901**	38	28.0	184.4	1.4	12.0	125.0	194.7	1802.9	34.8	191.6	2057.5
**1901**	53	21.4	158.0	1.2	10.0	104.6	139.8	1648.1	28.7	167.2	1864.3
**1901**	75	18.5	122.7	1.1	8.0	76.7	126.8	1455.4	22.8	147.9	1645.3
**1901**	106	17.5	124.1	1.0	7.6	70.2	104.1	1162.1	21.6	146.4	1298.1
**1904**	<38	42.7	245.9	1.4	17.5	148.5	532.5	766.2	53.7	1356.2	746.7
**1904**	38	30.0	159.7	0.8	12.9	109.6	215.3	557.3	35.2	1133.5	571.4
**1904**	53	26.8	128.9	0.8	10.5	88.9	140.6	466.7	29.0	1012.8	477.5
**1904**	75	22.6	108.7	0.6	9.0	70.5	249.5	390.4	23.9	803.0	415.8
**1904**	106	16.6	94.3	2.6	7.2	58.3	49.2	318.3	19.4	623.3	315.6
**1906**	<38	60.4	358.4	2.6	22.2	118.4	695.7	930.8	70.0	304.2	688.9
**1906**	38	41.5	245.7	1.8	15.9	95.4	212.6	799.6	47.8	213.1	571.7
**1906**	53	32.4	204.7	1.6	13.3	77.6	136.2	596.7	39.1	173.4	432.0
**1906**	75	25.7	160.1	1.5	10.3	64.6	81.4	430.7	29.2	140.3	294.2
**1906**	106	21.8	136.1	0.9	8.5	54.4	72.0	339.0	25.5	147.0	231.9
**1907**	<38	62.6	580.0	1.2	23.5	107.8	775.8	755.3	79.8	311.8	850.3
**1907**	38	45.4	385.6	0.2	17.2	82.2	235.0	629.3	57.8	225.9	625.5
**1907**	53	37.8	313.0	0.2	15.3	65.7	138.1	523.4	47.0	189.6	532.0
**1907**	75	29.8	237.9	0.1	11.0	52.7	77.0	443.0	34.8	164.2	434.4
**1907**	106	25.9	201.3	0.1	9.4	40.4	60.1	388.7	29.9	129.2	358.0

ANOVA analysis showed significant differences for the Pb (P < 0.001), Zn (P < 0.001), Mn (P < 0.001), Cd (P < 0.018), and Ba (P < 0.026) values in the four wells, indicating that the data sets were not normally distributed (Table S2). The LSD tests (Table S3) demonstrated that the Pb concentration in well 1904 was significantly higher than those in wells 1901, 1906, and 1907 (P < 0.001). The Zn concentration in well 1901 was significantly higher than those in wells 1904, 1906, and 1907 (P < 0.001). The Mn for well 1901 was significantly higher than those for wells 1904, 1906, and 1907 (P < 0.001). The Cd concentration in well 1901 was significantly higher than that in well 1907 (P = 0.020). The Cd concentration in well 1904 was significantly higher than that in well 1907 (P = 0.040). The Cd concentration in well 1906 was significantly higher than that in well 1907 (P = 0.003). In addition, the Ba concentration in well 1907 was significantly higher than those in wells 1901 (P = 0.013) and 1904 (P = 0.006).

**Figure 2:**
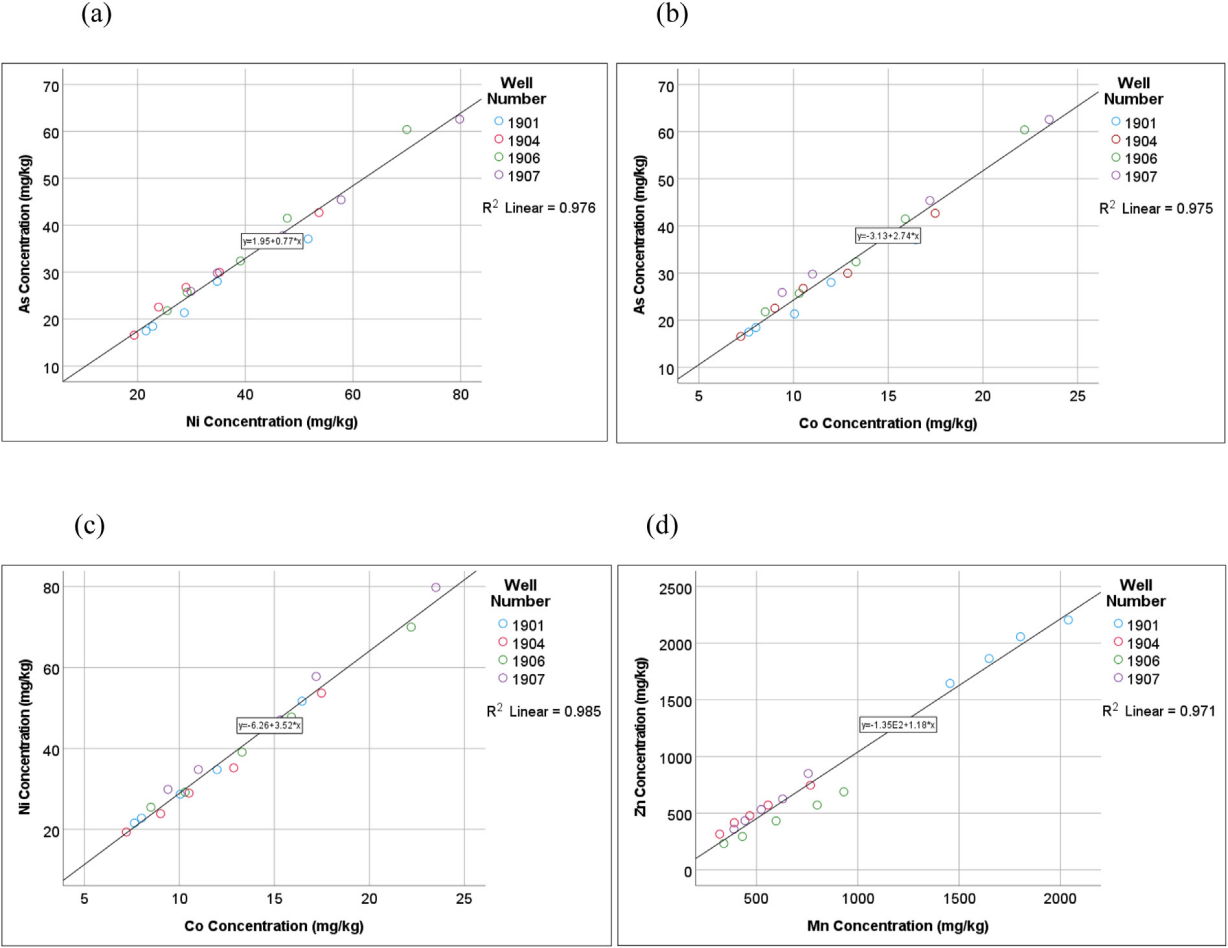
Heavy metals correlated using linear regression, (a) Correlation between As and Ni, (b) correlation between As and Co, (c) correlation between Ni and Co, (d) correlation between Zn and Mn.

Pearson's correlation analysis (2-tailed) showed significant positive correlations between As and Ba (r = 0.917, P < 0.001), As and Ni (r = 0.988, P < 0.001), As and Cr (r = 0.466, P = 0.038), As and Co (0.987, P < 0.001), and As and Cu (r = 0.846, P < 0.001). In addition, there were significant positive correlations between Zn and Mn (r = 0.986, P < 0.001), Zn and Cr (r = 0.620, P = 0.004), Mn and Cr (r = 0.668, P = 0.001), Cd and Cr (r = 0.465, P = 0.039), Ba and Ni (r = 0.942, P < 0.001), Ba and Co (r = 0.903, P < 0.001), Ba and Cu (r = 0.743, P < 0.001), Ni and Cr (r = 0.529, P = 0.017), Ni and Co (r = 0.993, P < 0.001), Ni and Cu (r = 0.862, P < 0.001), Cr and Co (r = 0.579, P = 0.008), Cr and Cu (r = 0.705, P = 0.001), and Co and Cu (r = 0.872, P < 0.001) (Table 2). Some of these correlations are illustrated in Figure 2 (a-d). The significant correlations between these heavy metals suggest their common origins and sinks in the MSW. They can be subsequently used to compare different environmental compartments around the landfill site, as the studied landfill has been previously assessed for LFM feasibility.

**Table 2:** Correlation matrix for heavy metals using Pearson's correlation (2-tailed) analysis.

Correlation matrix											
	As	Ba	Cd	Co	Cr	Cu	Mn	Ni	Pb	Zn	
As	Pearson Correlation	1	.917∗∗	0.189	.987∗∗	.466∗	.846∗∗	0.007	.988∗∗	0.039	−0.069
	Sig. (2-tailed)		0.000	0.424	0.000	0.038	0.000	0.976	0.000	0.870	0.774
	N	20	20	20	20	20	20	20	20	20	20
Ba	Pearson Correlation	.917∗∗	1	−0.006	.903∗∗	0.316	.743∗∗	0.037	.942∗∗	−0.166	0.006
	Sig. (2-tailed)	0.000		0.979	0.000	0.174	0.000	0.878	0.000	0.484	0.981
	N	20	20	20	20	20	20	20	20	20	20
Cd	Pearson Correlation	0.189	−0.006	1	0.238	.470∗	0.387	0.325	0.189	0.040	0.228
	Sig. (2-tailed)	0.424	0.979		0.312	0.036	0.091	0.162	0.426	0.868	0.334
	N	20	20	20	20	20	20	20	20	20	20
Co	Pearson Correlation	.987∗∗	.903∗∗	0.238	1	.579∗∗	.873∗∗	0.114	.993∗∗	0.074	0.039
	Sig. (2-tailed)	0.000	0.000	0.312		0.007	0.000	0.634	0.000	0.756	0.870
	N	20	20	20	20	20	20	20	20	20	20
Cr	Pearson Correlation	.466∗	0.316	.470∗	.579∗∗	1	.705∗∗	.668∗∗	.529∗	0.348	.620∗∗
	Sig. (2-tailed)	0.038	0.174	0.036	0.007		0.001	0.001	0.017	0.133	0.004
	N	20	20	20	20	20	20	20	20	20	20
Cu	Pearson Correlation	.846∗∗	.743∗∗	0.387	.873∗∗	.705∗∗	1	0.261	.862∗∗	0.231	0.210
	Sig. (2-tailed)	0.000	0.000	0.091	0.000	0.001		0.266	0.000	0.328	0.375
	N	20	20	20	20	20	20	20	20	20	20
Mn	Pearson Correlation	0.007	0.037	0.325	0.114	.668∗∗	0.261	1	0.103	−0.255	.986∗∗
	Sig. (2-tailed)	0.976	0.878	0.162	0.634	0.001	0.266		0.666	0.279	0.000
	N	20	20	20	20	20	20	20	20	20	20
Ni	Pearson Correlation	.988∗∗	.942∗∗	0.189	.993∗∗	.529∗	.862∗∗	0.103	1	0.010	0.036
	Sig. (2-tailed)	0.000	0.000	0.426	0.000	0.017	0.000	0.666		0.966	0.881
	N	20	20	20	20	20	20	20	20	20	20
Pb	Pearson Correlation	0.039	−0.166	0.040	0.074	0.348	0.231	−0.255	0.010	1	−0.220
	Sig. (2-tailed)	0.870	0.484	0.868	0.756	0.133	0.328	0.279	0.966		0.351
	N	20	20	20	20	20	20	20	20	20	20
Zn	Pearson Correlation	−0.069	0.006	0.228	0.039	.620∗∗	0.210	.986∗∗	0.036	−0.220	1
	Sig. (2-tailed)	0.774	0.981	0.334	0.870	0.004	0.375	0.000	0.881	0.351	
	N	20	20	20	20	20	20	20	20	20	20

∗∗Correlation is significant at the 0.01 level (2-tailed).

∗Correlation is significant at the 0.05 level (2-tailed).

It was clear that the concentrations of heavy metals increased with a decrease in the size of the waste fractions, with the values for As, Ba, Co, Cr, Cu, and Ni being significant (P < 0.02) according to Pearson's correlation (2-tailed) analysis, which supports previous findings. This richness of heavy metals in finer fractions is mainly due to the greater surface adsorption potential of heavy metals and ionic attraction compared to coarse particles, indicating a high specific surface area available for interaction (Filgueiras et al., 2002; Wei et al., 2015).

**Figure 3:**
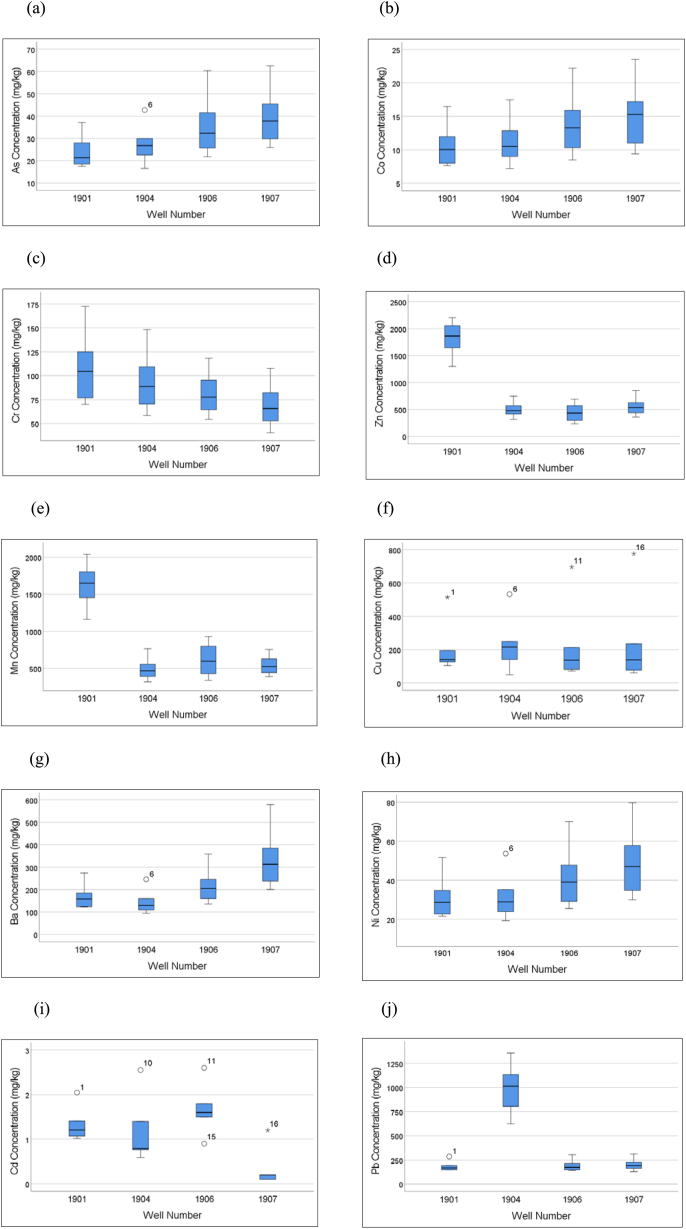
(a–j) Comparison of heavy metal concentrations from landfill samples recovered from the four wells, [(a) As, (b) Co, (c) Cr, (d) Zn, (e) Mn, (f) Cu, (g) Ba, (h) Ni, (i) Cd, (j) Pb]. The numbers above/below boxplots indicate which observation in the dataset (Table 1) is the outlier (numbers are arranged in descending order).

Figure 3(a–j) shows the concentrations of each heavy metal within the four wells of the analysed landfill samples. The box and whisker plots present the interquartile range (Q3–Q1), median (Q2, the line within the box), and outliers. The circle indicates that an outlier is present in the data, whereas the asterisk (∗) indicates that an extreme outlier is present in the data. The box plots reveal fluctuating heavy metal concentrations within the studied wells, indicating the heterogeneity of the MSW landfills. The availability of heavy metals in landfills is closely linked to site-specific landfill management operational practices, the nature of disposed waste, and degradation activities (Adelopo et al., 2018; Holm et al., 2002).

Compared to a previous study (Wagland et al., 2019) conducted on nine landfill sites in the UK, the levels of As, Pb, and Zn were much higher in the current study, whereas the Cd and Cu concentrations were relatively comparable. In contrast, significant accumulation of Cr was observed in the previous study (Wagland et al., 2019). Similarly, the Cu content in another study (Gutiérrez-Gutiérrez et al., 2015) was considerably higher than that in the current study.

Table 3 displays the generic assessment criteria of the UK SGVs based on the corresponding land use. SGVs only consider the assessment of human health risks originating from long-term on-site exposure to individual chemicals in soil (Environment Agency, 2004). Compared to recommended maximum allowable limits set by the UK Soil Guideline Values, the highest value of As exceeded the SGVs by 30.6 and 19.6 mg/kg for the residential and allotment land uses, respectively. In addition, the maximum concentrations of Cd and Cr were above the limits for the allotment and residential land uses, respectively, resulting in plant uptake. Similarly, the Cu and Zn levels were beyond the limits set for the allotment land use. The highest Pb concentration was considerably greater than the set limits for all land uses, except for the commercial land use. Concentrations of soil above the guideline levels may cause significant harm to human health (Nathanail et al., 2015).

**Table 3:** Descriptive statistics of heavy metals according to the generic Assessment Criteria of UK Soil Guideline Values.

Parameter	Range and mean (mg/kg) of analysed heavy metals	Function of Land Use	CLEA Soil Guideline Value (SGV) mg/kg	Reference
As	Max 62.58 Mean 32.20 Min 16.57	Residential Allotment Commercial	32 43 640	CL:AIRE (Environment Agency, 2009b)
Ni	Max 79.80 Mean 39.07 Min 19.36	Residential Allotment Commercial	130 230 1800	CL:AIRE (Environment Agency, 2009b)
Cd	Max 2.64 Mean 1.16 Min 0.09	Residential Allotment Commercial	10 1.8 230	CL:AIRE (Environment Agency, 2009b)
Cr	Max 172.75 Mean 89.20 Min 40.44	Residential with plant uptake Residential without plant uptake Commercial	130 200 5000	ALS (European Council, 2003)
Pb	Max 1356.16 Mean 393.28 Min 129.19	Residential with home grown- produce Residential without home grown produce Allotment Commercial	200 310 80 2300	ALS (European Council, 2003)
Cu	Max 775.82 Mean 237.52 Min 49.18	Allotment	520	LQM/CIEH S4Uls (Nathanail et al., 2015)
Zn	Max 2205.56 Mean 830.83 Min 231.94	Allotment	620	LQM/CIEH S4Uls (Nathanail et al., 2015)

#### 3.1.2 Total organic carbon (TOC)

**Figure 4:**
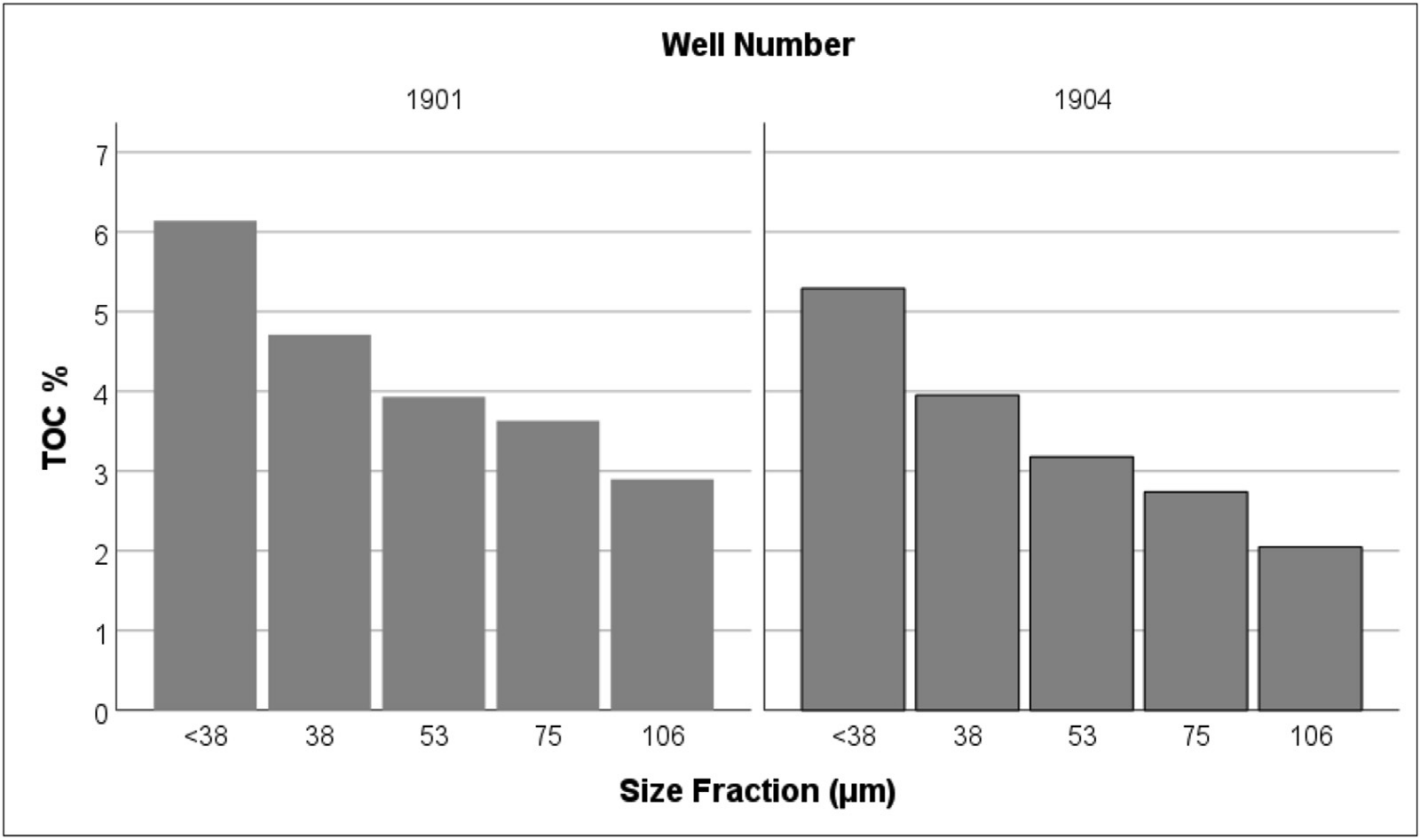
Total organic carbon values for different size fractions from wells 1901 and 1904.

The TOC results ranged from 1–6% (Figure 4), broadly consistent with the waste acceptance criterion (threshold) of 5% TOC for modern non-hazardous landfills in the UK under the landfill directive (European Council, 2003). With an average TOC of 1.5%, this categorises the case study site as an advanced methanogenic state landfill. A more significant amount of organic content is likely within the fine fractions of the excavated waste material, as degradation processes of organic waste decrease grain size fractions with the progression of time in landfills (Parrodi et al., 2018; Pecorini and Iannelli, 2020; Somani et al., 2018; Wei et al., 2015), depending on site-specific conditions. Hence, the TOC was analysed for fine fraction samples of ≤0.106 mm.

Pearson's correlation (2-tailed) showed a significant negative correlation between the size of waste fractions and TOC values (P < 0.01). This association is mainly due to the reduction in organic waste material particle size over time, which is promoted by biodegradation and weathering effects (Parrodi et al., 2018). Nevertheless, it is important to highlight that fine fractions within the landfill may also result from vertical transport in deeper layers, such as downward migration due to gravitational force. Nearly 70% of the total excavated waste fractions from the four drilled wells passed through a sieve diameter of 4.75 mm, while approximately 56% of the particles was <2.3 mm in size. These fractions were mainly soil-like materials with similar consistency to the soil. This result is consistent with previous research on the characterisation of excavated MSW samples (Parrodi et al., 2018; Quaghebeur et al., 2013; Wagland et al., 2019).

**Figure 5:**
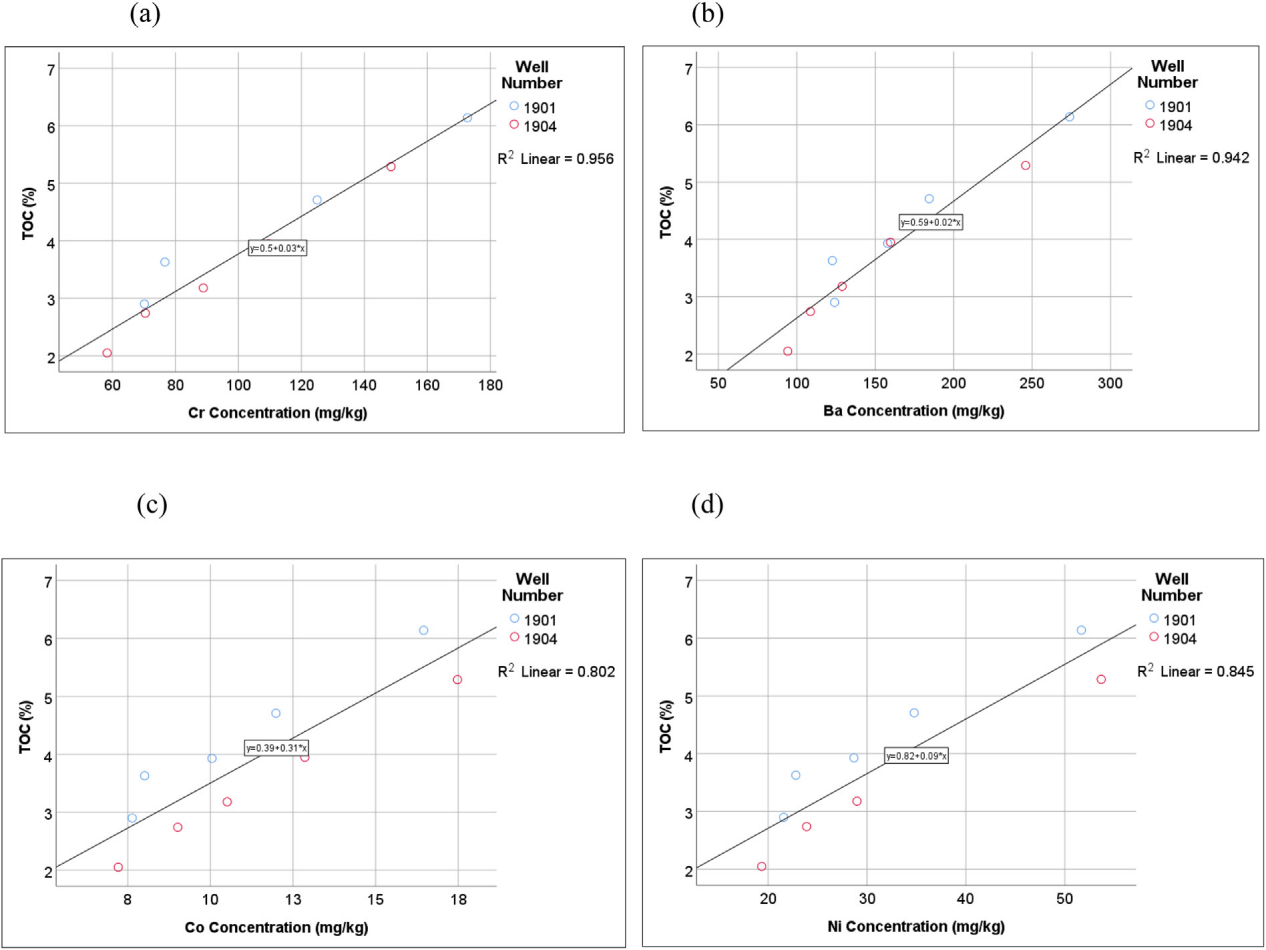
Correlation graph between total organic carbon and various heavy metals obtained using linear regression line, (a) correlation between TOC and Cr, (b) correlation between TOC and Ba, (c) correlation between TOC and Co, (d) correlation between TOC and Ni.

Pearson's correlation (2-tailed) also showed significant positive correlations between TOC and Ba (r = 0.971, P < 0.001), TOC and Cr (r = 0.978, P < 0.001), TOC and Ni (r = 0.919, P < 0.001), TOC and Co (r = 0.896, P < 0.001), TOC and As (r = 0.838, P < 0.003), and TOC and Cu (r = 0.834, P < 0.004). Some of the correlations are shown in Figure 5 (a–d).

The scientific reasoning behind the close association between organics and metals is that metals are likely to have been immobilised during waste degradation through a variety of processes, including sorption to soil particles and organic matter in the waste (Brand et al., 2018). Organic carbon is considered an essential elemental adsorbent in landfilled waste (Gutiérrez-Gutiérrez et al., 2015; Pandey et al., 2016; Wagland et al., 2019). Both humic and fulvic acids are the main components of organic materials and have a robust complexation capacity with heavy metals (Tang et al., 2014). Hence, more TOC is found within waste particles, and more heavy metals are adsorbed, thereby slowing the migration of heavy metals (Wei et al., 2015). Previous studies have demonstrated a significantly higher content of organic matter (Frank et al., 2017; García et al., 2016), which is explained by the difference in landfill management and status (Frank et al., 2017; Reinhart et al., 2002).

### 3.2 Pollution indicators

Only five of the ten heavy metals were considered for the geoaccumulation index and CF calculations because of the availability of established normal background concentration data for English soils. Table 4 shows the class distribution of the geoaccumulation index and the CFs of the heavy metals. Three of the heavy metals evaluated (As, Cd, and Ni) had geoaccumulation indices between zero and one, indicating no contamination to moderate contamination. The maximum value of Cu was above two (I_geo_ > 2/, indicating moderate to strong contamination), while its mean was 0.77, suggesting that some wells have a higher pollution potential than others, that is, contaminants are not uniformly distributed, as expected given the heterogeneity of landfilled waste. Similarly, the maximum value of Pb was >1, which indicates moderate contamination based on the classification categories.

**Table 4:** Results of the geoaccumulation index and the contamination factors of heavy metals.

Descriptive statistics (20 samples)	As	Cd	Cu	Ni	Pb
Geoaccumulation index (I_geo_)					
**Max.**	0.39	0.53	2.51	0.38	1.51
**Mean**	0.20	0.23	0.77	0.19	0.44
**Min.**	0.10	0.02	0.16	0.12	0.14
Contamination factor (CF)					
**Max.**	1.96	2.64	12.51	1.67	7.53
**Mean**	1.01	1.16	3.83	0.93	2.18
**Min.**	0.52	0.09	0.97	0.46	0.72

**Figure 6:**
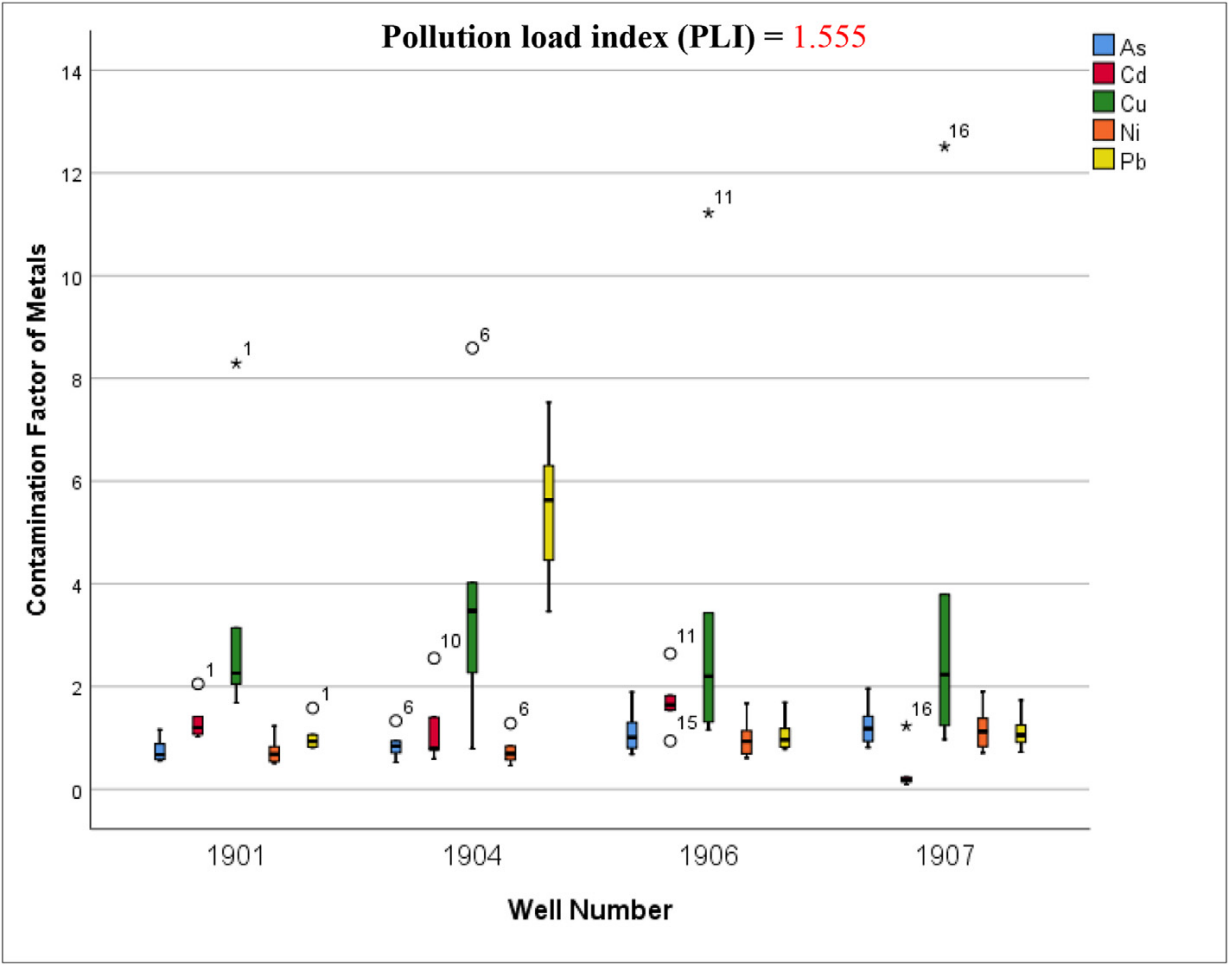
Boxplots of the contamination factor values of five heavy metals within the four wells.

The results of the CF revealed that the values followed a similar trend to the geoaccumulation index values, but with higher values owing to the direct calculation of the risk, which is different to how the geoaccumulation index was calculated. The CF values for As, Cd, and Ni fell between the categories of none-to-medium and moderate-to-strong. A significant concern was observed regarding the maximum CF values for Cu and Pb, with their pollution levels being classified as very strong (CF > 6). The mean CF values for Cu and Pb fell within the moderate-to-strong degree of contamination. Figure 6 illustrates the distribution pattern of the CF values within the four wells. The PLI value of 1.55 indicates that there is a pollution load within the site, which reflects the pollution of metals in waste materials. The nature of contamination found in the current study based on the three calculated indices is comparable to the results of previous investigations (Adelopo et al., 2018; Kolawole et al., 2018; Somani et al., 2020).

### 3.3 Health risk assessment

#### 3.3.1 Non-carcinogenic health hazard characterization

A total of seven out of ten heavy metals were considered in the non-carcinogenic assessment because Ba and Co do not yet have published SGVs. The calculation of the HQ was based on the individual land use, as SGVs are derived from different generic land use scenarios, which are described in detail in (Environment Agency, 2009a). The results of the HQ calculations are presented in Table 5. The highest HQ for As was 1.96 for the residential land use, followed by a value of 1.46 for the allotment land use. The mean for Pb was 4.92, with a maximum value of 16.95 for the allotment land use. The mean Zn value was greater than one for allotment land use. The HQ for Ni was the only metal within the acceptable limit (HQ < 1) for all land-use scenarios. High HQ values were observed, indicating heavy metal pollution that might pose non-cancer health risks to surrounding populations. Compared to the equations provided by the USEPA for the health risk assessment, the equation of HQ applied in this study is more suitable for this study because it uses the UK-based SGVs for different land uses.

**Table 5:** Potential human health non-carcinogenic risk assessment index (HQ) of heavy metals categorised by intended future land use.

Statistics (20 sample)	Hazard quotient (HQ)						
	As	Ni	Cd	Cr	Pb	Cu	Zn
Allotment							
**Maximum**	1.46	0.35	1.47		16.95	1.49	3.56
**Mean**	0.75	0.17	0.65		4.92	0.46	1.34
**Minimum**	0.39	0.08	0.05		1.61	0.09	0.37
Commercial							
**Maximum**	0.10	0.04	0.01	0.03	0.59		
**Mean**	0.05	0.02	0.01	0.02	0.17		
**Minimum**	0.03	0.01	0.00	0.01	0.06		
Residential							
**Maximum**	1.96	0.61	0.26				
**Mean**	1.01	0.30	0.12				
**Minimum**	0.52	0.15	0.01				
Residential with plant uptake/Residential without plant uptake							
**Maximum**				1.33/0.86			
**Mean**				0.69/0.45			
**Minimum**				0.31/0.20			
Residential with home grown-produce/Residential without home grown-produce							
**Maximum**					6.78/4.73		
**Mean**					1.97/1.27		
**Minimum**					0.65/0.42		

Following the results of the non-carcinogenic health risk assessment, the HI values are displayed in Table 6, with the categorised risk levels (Tenebe et al., 2018). The HI denotes the cumulative non-carcinogenic health risk index, and the highest mean value of HI was found for the allotment land use, followed by the residential land use. The heavy metal Pb was found to be the greatest contributor to non-carcinogenic risk.

**Table 6:** Overall potential for non-carcinogenic (HI) effects of heavy metals and the associated risk-level categories.

Descriptive	Hazard index (HI) mean values for non-carcinogenic risk	Level of risk
Allotment	8.28	High
Commercial	0.27	Low
Residential of all different uses	7.21	High

#### 3.3.2 Carcinogenic health risk analysis

**Figure 7:**
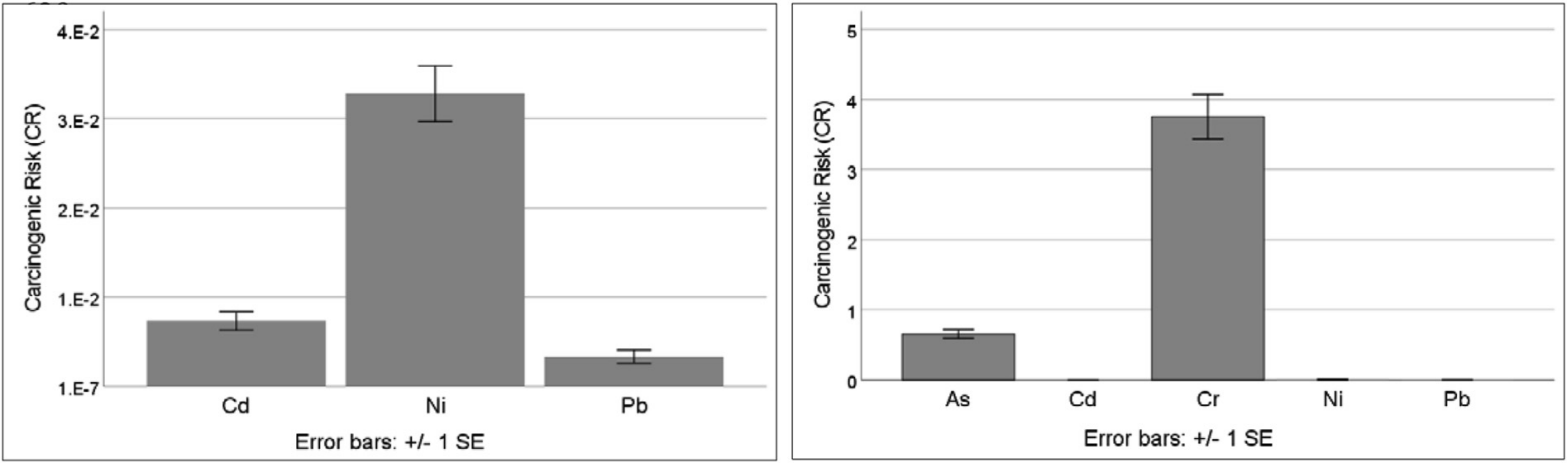
(a and b) Cancer risk (CR) values of heavy metals.

Owing to the lack of carcinogenic slope factors for Cu, Mn, Co, and Zn, only the cancer risks for the other five metals (As, Cd, Pb, Cr, and Ni) were estimated. The cancer risk values of the heavy metals are illustrated in Figure 7 (a and b), which presents a comparison between the elements. Overall, the CR values calculated to assess the carcinogenic health risk of metal (loid)s were found to be significantly higher than the acceptable range of 1.0 × 10^−6^ to 1.0 × 10^−4^. The CR factors from all routes implied that Cr and As were the most potent health risk hazards. The risk potential of the metals was in the order Cr > As > Cd > Pb > Ni, and the mean total LCR value was 4.44. Elevated values of cancer risk suggest that more attention should be paid to heavy metal concentrations prior to LFM, since heavy metals, including Cr, As, Cd, and Pb, are classified as metals with carcinogenic health risks (Doležalová Weissmannová et al., 2019). It is evident that, with respect to the carcinogenic risk levels and required standards, the values obtained indicate a risk to human health, especially in the case of Cr.

Compared to the current study, a recent study by (Wagland et al., 2019) showed significantly greater levels of chromium (834 mg/kg) within waste materials, suggesting the necessity to consider the human health risks posed by Cr in its enhanced landfill mining framework.

## 4 Conclusions

This research is the first to determine the concentration and extent of heavy metal pollution and the associated health and environmental risks, as inadequate attention has previously been paid to the potential health risks associated with LFM activities. Well-established statistical methods and environmental and health risk indices were used for the assessment. The concentrations of As, Cd, Cr, Pb, Cu, and Zn in the present study were above the permissible limits set for soil in the UK. The Zn and Pb concentrations were found to be the highest in wells 1901 and 1904, respectively, compared to established SGVs. The concentrations also varied significantly among the four wells and decreased in the following order: Zn > Mn > Pb > Cu > Ba > Cr > Ni > As > Co > Cd. Waste fractions of different sizes demonstrated similar behaviours in the four different wells; approximately 56% of the excavated waste materials were ≤2.3 mm in size. The results of the TOC analysis were within the waste acceptance criterion thresholds set by the Landfill Directive that applies to current UK landfills. Regarding the I_geo_ and CF values, the concentrations of heavy metals were in the following order: Cu > Pb > Cd > As > Ni. The pollution load index (PLI) was >1, indicating pollution. The study found that the landfill poses a major risk to human health if LFM operations were to occur, with the non-carcinogenic risks of Zn and Pb being higher than the levels set by the USEPA. The carcinogenic effect revealed that Cr was the most prominent metal, followed by As, which could impact human health.

This research presents a novel approach used to calculate and assess potential risks to human health in the application of LFM activities and reveals useful information that needs to be considered in policy development for the excavation, processing, and sustainable reuse of waste from LFM. The design and implementation of LFM processes must give adequate consideration to occupational health (protection of site workers), protection of human health off-site, and the surrounding environment to ensure safe working practice. The characterisation of MSW samples analysed here provides an indication of the components within the waste from a landfill site that is typical of those predominantly MSW sites in the UK that could be considered suitable for LFM. Potential impacts from identified risks, whether to the environment or to human health, could be mitigated through careful design of LFM activities to reduce these short-term episodic emissions. LFM site selection criteria need to be developed to allow landowners with multiple sites (land banks) to prioritise suitability of individual sites based on sound science rather than decisions necessarily being development or cost led. Extensive site-specific systematic sampling regimes, tied in to well drilling programmes, may confirm the presence of elevated heavy metal levels in those landfills being considered. The high concentrations of potentially toxic elements found in the present study suggests the need for air dispersion modelling to determine the impact of LFM activities on air quality. This study provides a basis for more detailed studies on environmental management of LFM. From an international scientific viewpoint, the findings of this research and the role of LFM on contaminant mobility into the wider environment might become highly significant in the coming decades owing to climate change and the increasing demand for land use, which can substantially increase the potential for dust emissions and transport.

## Declarations

### Author contribution statement

Mohammed Zari: Conceived and designed the experiments; Performed the experiments; Analyzed and interpreted the data; Wrote the paper.

Richard Smith: Conceived and designed the experiments; Analyzed and interpreted the data.

Rebecca Ferrari: Conceived and designed the experiments; Analyzed and interpreted the data; Contributed reagents, materials, analysis tools or data.

### Funding statement

This work was supported by King Abdulaziz University, Saudi Arabia.

### Data availability statement

Data will be made available on request.

### Declaration of interest's statement

The authors declare no conflict of interest.

### Additional information

No additional information is available for this paper.

## Figures and Tables

**Table A1:** Descriptive statistics of the heavy metals within the four wells.

Descriptive Statistics											
Label		As	Ba	Cd	Co	Cr	Cu	Mn	Ni	Pb	Zn
**1901**	Mean	24.48	172.63	1.35	10.83	109.85	215.90	1621.52	31.90	187.65	1814.14
	N	5	5	5	5	5	5	5	5	5	5
	Std. Deviation	8.16	62.17	0.42	3.60	41.50	170.02	334.18	12.24	57.48	356.99
**1904**	Mean	27.72	147.51	1.22	11.41	95.15	237.42	499.77	32.23	985.76	505.40
	N	5	5	5	5	5	5	5	5	5	5
	Std. Deviation	9.76	60.23	0.81	3.97	35.55	182.03	173.41	13.37	284.86	163.90
**1906**	Mean	36.36	221.00	1.68	14.04	82.08	239.58	619.36	42.32	195.60	443.74
	N	5	5	5	5	5	5	5	5	5	5
	Std. Deviation	15.38	87.57	0.61	5.37	25.44	261.04	247.08	17.76	67.11	189.64
**1907**	Mean	40.30	343.56	0.36	15.28	69.76	257.20	547.94	49.86	204.14	560.04
	N	5	5	5	5	5	5	5	5	5	5
	Std. Deviation	14.55	150.01	0.47	5.57	26.32	297.89	147.13	19.96	69.80	191.00
**Total**	Mean	32.21	221.17	1.15	12.89	89.21	237.52	822.15	39.08	393.29	830.83
	N	20	20	20	20	20	20	20	20	20	20
	Std. Deviation	13.08	117.98	0.74	4.71	33.84	215.21	522.84	16.69	378.09	623.52

## Appendix A Supplementary data

Supplementary materials.docx Supplementary materials.docx
