# Supplementary material for: Health and environmental impact assessment of landfill mining activities: A case study in Norfolk, UK
Source: Heliyon. 2022 Nov 17;8(11):e11594. doi: 10.1016/j.heliyon.2022.e11594 (PMC9678709; doi:10.1016/j.heliyon.2022.e11594)
Supplement: Supplementary materials.docx [file mmc1.docx]

**Health and Environmental Impact Assessment of Landfill Mining Activities: A Case Study in Norfolk, UK**

**Mohammed Zari ^a, b,^** **^*^ , Richard Smith ^a, c^, Charles Wright ^d^ and Rebecca Ferrari ^a^**

**^a^** University of Nottingham, Faculty of Engineering, Chemical and Environmental Engineering Department, Coates Building, University Park, Nottingham NG7 2RD, United Kingdom

^b^ Department of Environmental Science, Faculty of Meteorology, Environment and Arid Land Agriculture, King Abdulaziz University, Jeddah, Saudi Arabia

^c^ Industrial Chemicals Ltd, Titan Works, Hogg Lane, Grays, Essex RM17 5DU, United Kingdom

^d^ Norfolk County Council, County Hall, Martineau Ln, Norwich NR1 2DH, United Kingdom

* Author to whom correspondence should be addressed [Mohammed.Zari@nottingham.ac.uk](mailto:Mohammed.Zari@nottingham.ac.uk)

**Supplementary materials**

**Table S1.** Classification levels of geo-accumulation index (I_geo_) and contamination factor (CF) indices.

| Geo-accumulation index (I_geo_) | | | Contamination factor (Cn) | | |
| --- | --- | --- | --- | --- | --- |
| Class | **Value** | **Classification** | **Level** | **Value** | **Categorisation** |
| 0 | <0 | Uncontaminated | 0 | 0 | None |
| 1 | 0-1 | Uncontaminated to moderately contaminated | 1 | 1 | None to medium |
| 2 | 1-2 | Moderately contaminated | 2 | 2 | Moderate |
| 3 | 2-3 | Moderately to strongly contaminated | 3 | 3 | Moderate to strong, |
| 4 | 3-4 | Strongly contaminated | 4 | 4 | Strongly polluted |
| 5 | 4-5 | Strongly to extremely contaminated | 5 | 5 | Strong to very strong |
| 6 | >5 | Extremely contaminated | 6 | ≥6 | Very strong |

**Table S2.** One-Way ANOVA test of heavy metals for all wells.

| ANOVA | | | | | | |
| --- | --- | --- | --- | --- | --- | --- |
|  |  | **Sum of Squares** | **df** | **Mean Square** | **F** | **Sig.** |
| As | Between Groups | 812.819 | 3 | 270.94 | 1.776 | 0.192 |
|  | Within Groups | 2440.22 | 16 | 152.514 |  |  |
|  | Total | 3253.038 | 19 |  |  |  |
| Pb | Between Groups | 2340847.978 | 3 | 780282.659 | 33.267 | 0 |
|  | Within Groups | 375287.871 | 16 | 23455.492 |  |  |
|  | Total | 2716135.85 | 19 |  |  |  |
| Zn | Between Groups | 6479886.722 | 3 | 2159962.241 | 38.103 | 0 |
|  | Within Groups | 906997.289 | 16 | 56687.331 |  |  |
|  | Total | 7386884.011 | 19 |  |  |  |
| Mn | Between Groups | 4296176.35 | 3 | 1432058.783 | 25.522 | 0 |
|  | Within Groups | 897769.119 | 16 | 56110.57 |  |  |
|  | Total | 5193945.469 | 19 |  |  |  |
| Cd | Between Groups | 4.748 | 3 | 1.583 | 4.446 | 0.019 |
|  | Within Groups | 5.695 | 16 | 0.356 |  |  |
|  | Total | 10.443 | 19 |  |  |  |
| Ba | Between Groups | 113805.749 | 3 | 37935.25 | 4.029 | 0.026 |
|  | Within Groups | 150652.146 | 16 | 9415.759 |  |  |
|  | Total | 264457.895 | 19 |  |  |  |
| Ni | Between Groups | 1125.754 | 3 | 375.251 | 1.44 | 0.268 |
|  | Within Groups | 4168.895 | 16 | 260.556 |  |  |
|  | Total | 5294.649 | 19 |  |  |  |
| Cr | Between Groups | 4452.114 | 3 | 1484.038 | 1.372 | 0.287 |
|  | Within Groups | 17303.757 | 16 | 1081.485 |  |  |
|  | Total | 21755.871 | 19 |  |  |  |
| Co | Between Groups | 67.348 | 3 | 22.449 | 1.014 | 0.412 |
|  | Within Groups | 354.298 | 16 | 22.144 |  |  |
|  | Total | 421.646 | 19 |  |  |  |
| Cu | Between Groups | 4295.221 | 3 | 1431.74 | 0.026 | 0.994 |
|  | Within Groups | 875662.96 | 16 | 54728.935 |  |  |
|  | Total | 879958.181 | 19 |  |  |  |

**Table S3.** Multiple comparisons analysis between heavy metal values of the four wells.

| Multiple Comparisons  LSD 95% Confidence Interval | | | | | | | |
| --- | --- | --- | --- | --- | --- | --- | --- |
| Dependent Variable | (I) Label | (J) Label | Mean Difference (I-J) | Std. Error | Sig. | Lower Bound/Upper Bound | |
| As | 1901 | 1904 | -3.23168 | 7.8106 | 0.685 | -19.7894 | 13.3261 |
|  |  | 1906 | -11.87613 | 7.8106 | 0.148 | -28.4339 | 4.6816 |
|  |  | 1907 | -15.81613 | 7.8106 | 0.06 | -32.3739 | 0.7416 |
|  | 1904 | 1901 | 3.23168 | 7.8106 | 0.685 | -13.3261 | 19.7894 |
|  |  | 1906 | -8.64445 | 7.8106 | 0.285 | -25.2022 | 7.9133 |
|  |  | 1907 | -12.58445 | 7.8106 | 0.127 | -29.1422 | 3.9733 |
|  | 1906 | 1901 | 11.87613 | 7.8106 | 0.148 | -4.6816 | 28.4339 |
|  |  | 1904 | 8.64445 | 7.8106 | 0.285 | -7.9133 | 25.2022 |
|  |  | 1907 | -3.94 | 7.8106 | 0.621 | -20.4977 | 12.6177 |
|  | 1907 | 1901 | 15.81613 | 7.8106 | 0.06 | -0.7416 | 32.3739 |
|  |  | 1904 | 12.58445 | 7.8106 | 0.127 | -3.9733 | 29.1422 |
|  |  | 1906 | 3.94 | 7.8106 | 0.621 | -12.6177 | 20.4977 |
| Pb | 1901 | 1904 | -798.11263* | 96.86174 | 0 | -1003.4503 | -592.775 |
|  |  | 1906 | -7.9514 | 96.86174 | 0.936 | -213.2891 | 197.3863 |
|  |  | 1907 | -16.4914 | 96.86174 | 0.867 | -221.8291 | 188.8463 |
|  | 1904 | 1901 | 798.11263* | 96.86174 | 0 | 592.7749 | 1003.45 |
|  |  | 1906 | 790.16122* | 96.86174 | 0 | 584.8235 | 995.4989 |
|  |  | 1907 | 781.62122* | 96.86174 | 0 | 576.2835 | 986.9589 |
|  | 1906 | 1901 | 7.9514 | 96.86174 | 0.936 | -197.3863 | 213.2891 |
|  |  | 1904 | -790.16122* | 96.86174 | 0 | -995.4989 | -584.824 |
|  |  | 1907 | -8.54 | 96.86174 | 0.931 | -213.8777 | 196.7977 |
|  | 1907 | 1901 | 16.4914 | 96.86174 | 0.867 | -188.8463 | 221.8291 |
|  |  | 1904 | -781.62122* | 96.86174 | 0 | -986.9589 | -576.284 |
|  |  | 1906 | 8.54 | 96.86174 | 0.931 | -196.7977 | 213.8777 |
| Zn | 1901 | 1904 | 1308.74606* | 150.58198 | 0 | 989.5265 | 1627.966 |
|  |  | 1906 | 1370.40324* | 150.58198 | 0 | 1051.1837 | 1689.623 |
|  |  | 1907 | 1254.10324* | 150.58198 | 0 | 934.8837 | 1573.323 |
|  | 1904 | 1901 | -1308.74606* | 150.58198 | 0 | -1627.9656 | -989.527 |
|  |  | 1906 | 61.65719 | 150.58198 | 0.688 | -257.5623 | 380.8767 |
|  |  | 1907 | -54.64281 | 150.58198 | 0.721 | -373.8623 | 264.5767 |
|  | 1906 | 1901 | -1370.40324* | 150.58198 | 0 | -1689.6228 | -1051.18 |
|  |  | 1904 | -61.65719 | 150.58198 | 0.688 | -380.8767 | 257.5623 |
|  |  | 1907 | -116.3 | 150.58198 | 0.451 | -435.5195 | 202.9195 |
|  | 1907 | 1901 | -1254.10324* | 150.58198 | 0 | -1573.3228 | -934.884 |
|  |  | 1904 | 54.64281 | 150.58198 | 0.721 | -264.5767 | 373.8623 |
|  |  | 1906 | 116.3 | 150.58198 | 0.451 | -202.9195 | 435.5195 |
| Mn | 1901 | 1904 | 1121.74715* | 149.81398 | 0 | 804.1557 | 1439.339 |
|  |  | 1906 | 1002.16098* | 149.81398 | 0 | 684.5695 | 1319.752 |
|  |  | 1907 | 1073.58098* | 149.81398 | 0 | 755.9895 | 1391.172 |
|  | 1904 | 1901 | -1121.74715* | 149.81398 | 0 | -1439.3386 | -804.156 |
|  |  | 1906 | -119.58617 | 149.81398 | 0.436 | -437.1776 | 198.0053 |
|  |  | 1907 | -48.16616 | 149.81398 | 0.752 | -365.7576 | 269.4253 |
|  | 1906 | 1901 | -1002.16098* | 149.81398 | 0 | -1319.7524 | -684.57 |
|  |  | 1904 | 119.58617 | 149.81398 | 0.436 | -198.0053 | 437.1776 |
|  |  | 1907 | 71.42 | 149.81398 | 0.64 | -246.1714 | 389.0114 |
|  | 1907 | 1901 | -1073.58098* | 149.81398 | 0 | -1391.1724 | -755.99 |
|  |  | 1904 | 48.16616 | 149.81398 | 0.752 | -269.4253 | 365.7576 |
|  |  | 1906 | -71.42 | 149.81398 | 0.64 | -389.0114 | 246.1714 |
| Cd | 1901 | 1904 | 0.13091 | 0.37733 | 0.733 | -0.669 | 0.9308 |
|  |  | 1906 | -0.33021 | 0.37733 | 0.394 | -1.1301 | 0.4697 |
|  |  | 1907 | .98979* | 0.37733 | 0.018 | 0.1899 | 1.7897 |
|  | 1904 | 1901 | -0.13091 | 0.37733 | 0.733 | -0.9308 | 0.669 |
|  |  | 1906 | -0.46112 | 0.37733 | 0.239 | -1.261 | 0.3388 |
|  |  | 1907 | .85888* | 0.37733 | 0.037 | 0.059 | 1.6588 |
|  | 1906 | 1901 | 0.33021 | 0.37733 | 0.394 | -0.4697 | 1.1301 |
|  |  | 1904 | 0.46112 | 0.37733 | 0.239 | -0.3388 | 1.261 |
|  |  | 1907 | 1.32000* | 0.37733 | 0.003 | 0.5201 | 2.1199 |
|  | 1907 | 1901 | -.98979* | 0.37733 | 0.018 | -1.7897 | -0.1899 |
|  |  | 1904 | -.85888* | 0.37733 | 0.037 | -1.6588 | -0.059 |
|  |  | 1906 | -1.32000* | 0.37733 | 0.003 | -2.1199 | -0.5201 |
| Ba | 1901 | 1904 | 25.11442 | 61.37022 | 0.688 | -104.9846 | 155.2135 |
|  |  | 1906 | -48.37288 | 61.37022 | 0.442 | -178.4719 | 81.7262 |
|  |  | 1907 | -170.93288* | 61.37022 | 0.013 | -301.0319 | -40.8338 |
|  | 1904 | 1901 | -25.11442 | 61.37022 | 0.688 | -155.2135 | 104.9846 |
|  |  | 1906 | -73.4873 | 61.37022 | 0.249 | -203.5863 | 56.6118 |
|  |  | 1907 | -196.04730* | 61.37022 | 0.006 | -326.1463 | -65.9482 |
|  | 1906 | 1901 | 48.37288 | 61.37022 | 0.442 | -81.7262 | 178.4719 |
|  |  | 1904 | 73.4873 | 61.37022 | 0.249 | -56.6118 | 203.5863 |
|  |  | 1907 | -122.56 | 61.37022 | 0.063 | -252.6591 | 7.5391 |
|  | 1907 | 1901 | 170.93288* | 61.37022 | 0.013 | 40.8338 | 301.0319 |
|  |  | 1904 | 196.04730* | 61.37022 | 0.006 | 65.9482 | 326.1463 |
|  |  | 1906 | 122.56 | 61.37022 | 0.063 | -7.5391 | 252.6591 |
| Ni | 1901 | 1904 | -0.32586 | 10.20894 | 0.975 | -21.9678 | 21.3161 |
|  |  | 1906 | -10.41691 | 10.20894 | 0.323 | -32.0589 | 11.2251 |
|  |  | 1907 | -17.95691 | 10.20894 | 0.098 | -39.5989 | 3.6851 |
|  | 1904 | 1901 | 0.32586 | 10.20894 | 0.975 | -21.3161 | 21.9678 |
|  |  | 1906 | -10.09105 | 10.20894 | 0.338 | -31.733 | 11.5509 |
|  |  | 1907 | -17.63105 | 10.20894 | 0.103 | -39.273 | 4.0109 |
|  | 1906 | 1901 | 10.41691 | 10.20894 | 0.323 | -11.2251 | 32.0589 |
|  |  | 1904 | 10.09105 | 10.20894 | 0.338 | -11.5509 | 31.733 |
|  |  | 1907 | -7.54 | 10.20894 | 0.471 | -29.182 | 14.102 |
|  | 1907 | 1901 | 17.95691 | 10.20894 | 0.098 | -3.6851 | 39.5989 |
|  |  | 1904 | 17.63105 | 10.20894 | 0.103 | -4.0109 | 39.273 |
|  |  | 1906 | 7.54 | 10.20894 | 0.471 | -14.102 | 29.182 |
| Cr | 1901 | 1904 | 14.7036 | 20.79889 | 0.49 | -29.3881 | 58.7953 |
|  |  | 1906 | 27.77062 | 20.79889 | 0.2 | -16.3211 | 71.8623 |
|  |  | 1907 | 40.09062 | 20.79889 | 0.072 | -4.0011 | 84.1823 |
|  | 1904 | 1901 | -14.7036 | 20.79889 | 0.49 | -58.7953 | 29.3881 |
|  |  | 1906 | 13.06702 | 20.79889 | 0.539 | -31.0247 | 57.1587 |
|  |  | 1907 | 25.38702 | 20.79889 | 0.24 | -18.7047 | 69.4787 |
|  | 1906 | 1901 | -27.77062 | 20.79889 | 0.2 | -71.8623 | 16.3211 |
|  |  | 1904 | -13.06702 | 20.79889 | 0.539 | -57.1587 | 31.0247 |
|  |  | 1907 | 12.32 | 20.79889 | 0.562 | -31.7717 | 56.4117 |
|  | 1907 | 1901 | -40.09062 | 20.79889 | 0.072 | -84.1823 | 4.0011 |
|  |  | 1904 | -25.38702 | 20.79889 | 0.24 | -69.4787 | 18.7047 |
|  |  | 1906 | -12.32 | 20.79889 | 0.562 | -56.4117 | 31.7717 |
| Co | 1901 | 1904 | -0.58816 | 2.97615 | 0.846 | -6.8973 | 5.721 |
|  |  | 1906 | -3.21356 | 2.97615 | 0.296 | -9.5227 | 3.0956 |
|  |  | 1907 | -4.45356 | 2.97615 | 0.154 | -10.7627 | 1.8556 |
|  | 1904 | 1901 | 0.58816 | 2.97615 | 0.846 | -5.721 | 6.8973 |
|  |  | 1906 | -2.62539 | 2.97615 | 0.391 | -8.9345 | 3.6838 |
|  |  | 1907 | -3.86539 | 2.97615 | 0.212 | -10.1745 | 2.4438 |
|  | 1906 | 1901 | 3.21356 | 2.97615 | 0.296 | -3.0956 | 9.5227 |
|  |  | 1904 | 2.62539 | 2.97615 | 0.391 | -3.6838 | 8.9345 |
|  |  | 1907 | -1.24 | 2.97615 | 0.682 | -7.5491 | 5.0691 |
|  | 1907 | 1901 | 4.45356 | 2.97615 | 0.154 | -1.8556 | 10.7627 |
|  |  | 1904 | 3.86539 | 2.97615 | 0.212 | -2.4438 | 10.1745 |
|  |  | 1906 | 1.24 | 2.97615 | 0.682 | -5.0691 | 7.5491 |
| Cu | 1901 | 1904 | -21.52163 | 147.95801 | 0.886 | -335.1786 | 292.1353 |
|  |  | 1906 | -23.68148 | 147.95801 | 0.875 | -337.3385 | 289.9755 |
|  |  | 1907 | -41.30148 | 147.95801 | 0.784 | -354.9585 | 272.3555 |
|  | 1904 | 1901 | 21.52163 | 147.95801 | 0.886 | -292.1353 | 335.1786 |
|  |  | 1906 | -2.15984 | 147.95801 | 0.989 | -315.8168 | 311.4971 |
|  |  | 1907 | -19.77984 | 147.95801 | 0.895 | -333.4368 | 293.8771 |
|  | 1906 | 1901 | 23.68148 | 147.95801 | 0.875 | -289.9755 | 337.3385 |
|  |  | 1904 | 2.15984 | 147.95801 | 0.989 | -311.4971 | 315.8168 |
|  |  | 1907 | -17.62 | 147.95801 | 0.907 | -331.277 | 296.037 |
|  | 1907 | 1901 | 41.30148 | 147.95801 | 0.784 | -272.3555 | 354.9585 |
|  |  | 1904 | 19.77984 | 147.95801 | 0.895 | -293.8771 | 333.4368 |
|  |  | 1906 | 17.62 | 147.95801 | 0.907 | -296.037 | 331.277 |
| * The mean difference is significant at the 0.05 level. | | | | | | | |
